# Daily actions, challenges, and needs among Dutch parents while supporting the participation of their child with a physical disability at home, at school, and in the community: a qualitative diary study

**DOI:** 10.1186/s12887-016-0768-6

**Published:** 2017-01-11

**Authors:** Barbara Piškur, Anna J. H. M. Beurskens, Marjolijn Ketelaar, Marian J. Jongmans, Barbara M. Casparie, Rob J. E. M. Smeets

**Affiliations:** 1 Research Centre Autonomy and Participation for Persons with a Chronic Illness, Faculty of Health, Zuyd University of Applied Science, Nieuw Eyckholt 300, 6419 DJ Heerlen, The Netherlands; 2 Department of Occupational Therapy, Faculty of Health, Zuyd University of Applied Science, Nieuw Eyckholt 300, 6419 DJ Heerlen, The Netherlands; 3 Department of Rehabilitation Medicine, CAPHRI, School for Public Health and Primary Care, Faculty of Health, Medicine and Life Sciences, Maastricht University, P.O. Box 616, 6200 MD Maastricht, The Netherlands; 4 Department of General Practice, CAPHRI, School for Public Health and Primary Care, Faculty of Health, Medicine and Life Sciences, Maastricht University, P.O. Box 616, 6200 MD Maastricht, The Netherlands; 5 Brain Centre Rudolf Magnus and Centre of Excellence for Rehabilitation Medicine, University Medical Centre Utrecht and De Hoogstraat Rehabilitation, Rembrandtkade 10, 3583 TM Utrecht, The Netherlands; 6 Partner of NetChild, University Network for Childhood Disability Research in the Netherlands, Utrecht, The Netherlands; 7 Department of Child, Family and Education Studies, Faculty of Social and Behavioral Sciences, Utrecht University, PO Box 80140, 3508 TC Utrecht, The Netherlands; 8 Department of Neonatology, Wilhemina Children’s Hospital, University Medical Centre Utrecht, AB 3508 Utrecht, The Netherlands; 9 BOSK, The Dutch Association of People with Disabilities and Their Parents, 3502 GJ Utrecht, The Netherlands; 10 Adelante Centre of Expertise in Rehabilitation, Zandbergsweg 111, 6432 CC Hoensbroek, The Netherlands

**Keywords:** Children, Physical disability, Parents, Actions, Challenges, Needs, Qualitative study, Diary method, Family centred service, Environment

## Abstract

**Background:**

Parents have a vital influence on the participation of their child with a physical disability. The aim of this study is to gain insight into parents’ own daily actions, challenges, and needs while supporting their child with a physical disability at home, at school, and in the community. An additional objective of this study is to refine the preliminary thematic framework previously identified in a scoping review.

**Methods:**

A qualitative research inquiry was performed based on using a diary over a 7-day period to gather data. To systematically organise data into a structured format, content analysis has been applied using both inductive and deductive reasoning guided by the existing preliminary thematic framework.

**Results:**

Analysis of the eligible diaries shows that the actions mentioned by the 47 parents describe several efforts to enhance participation of their children with a physical disability by using, enabling, or changing the social and physical environment, or by supporting their child to perform or engage in meaningful activities. Those parents’ actions are primarily a result of challenges caused by restrictions in social and physical environments. Parental responses highlighted, above all, the need for environments designed for all people. Based on the findings a redefined thematic framework is presented.

**Conclusions:**

Parents’ actions, challenges, and needs are mainly directed towards the social or/and physical environment. The presented thematic framework can offer practitioners knowledge to support parents. More work is necessary to provide tailored approaches. Paediatric rehabilitation may need to address the importance of the environment on the participation of a child with a physical disability.

## Background

Parents play a significant role in enabling participation of children with a physical disability at home, at school, and in the community. Participation — the involvement in life situations to fulfil social roles — has a positive impact on children’s health and well-being [1–3]. Children with a physical disability often come across restrictions in their everyday participation [4–6]. The most important factor influencing successful participation of children and adolescents with disabilities is the interplay between the child’s environment and activities [7–12]. Parents have knowledge and experiences how to involve child’s social environment (e.g. peers) to support engagement in preferred and desired activities [13].

**Fig. 1:**
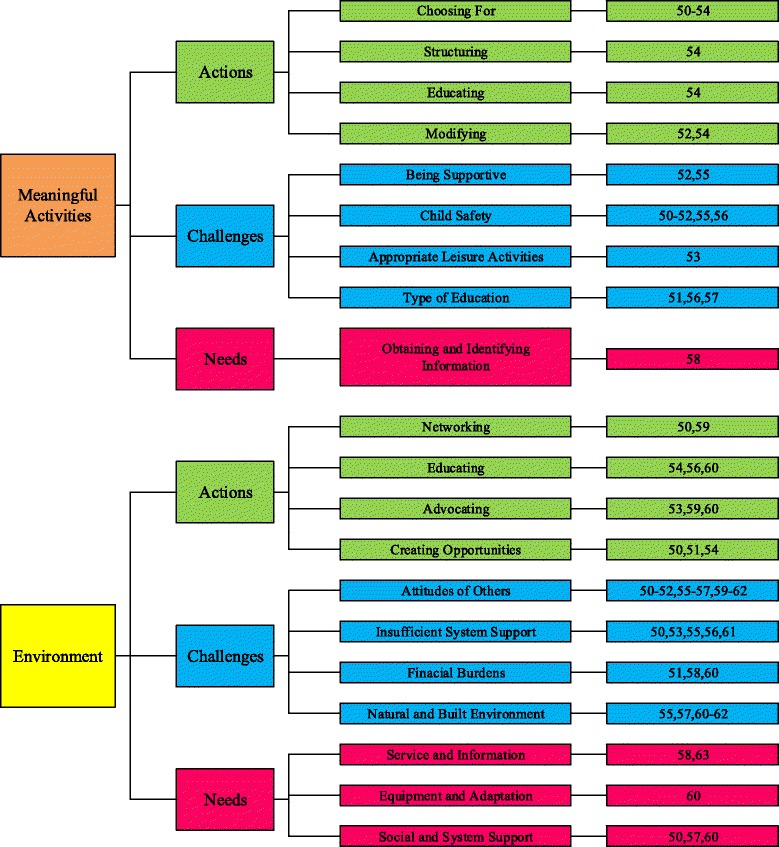
A preliminary thematic framework [17]

Family-Centred Service (FCS) is considered best practice in service for children with a physical disability [14]. Its effectiveness is related to the understanding professionals have about needs and actions of both child and parents, including what parents do to support their child with a physical disability [15, 16]. A recent scoping review of the literature on this topic [17] revealed 14 studies, which identified several parents’ actions, challenges, and needs that were summarised in a preliminary thematic framework. The framework includes two major themes: “parents enable and support performance of meaningful activities of their child at home, at school and in the community” and “parents enable, change and use the environment” [17]. Connected to the major themes, the framework includes three categories (actions, challenges, and needs) and several subcategories with a total of eight actions, eight challenges, and four needs, as presented in Fig. 1.

However, the scoping review [17] also underlines how little information is available on what parents actually do every day to enhance their child’s participation. Time-use diaries are particularly relevant and suitable instruments for studying the daily lives of families, including families with a child with a physical disability, as they give insight into what they as a family actually do in context [18]. Participants who fill out the diaries are both observers and informants, providing the researcher a “view from within” [19, 20].

The aim of this study is to gain insight into parents’ own daily actions, challenges, and needs while supporting their child with a physical disability at home, at school, and in the community. Additionally, the study results will be used to refine the preliminary thematic framework.

## Methods

Between May 2012 and July 2012, a qualitative research inquiry was performed based on using a diary over a 7-day period. Direct content analysis was used as theoretical framework to systematically organise data into a structured format [21–23].

### Study population

Potential participant families of children with a physical disability were selected from the electronic database of the Dutch Association of People with Disabilities and their Parents (BOSK). All potential families had a child aged between 4 and 12 years with a physical disability that is neurological and non-progressive in nature (e.g. cerebral palsy, spina bifida) and who was living at home. Persons identified in the database of the BOSK as the contact person for each family (parent or primary caregiver) were considered eligible for this study; in total 47 parents participated in this study.

### Recruitment procedure

In May 2012, 559 (17.2% from all the BOSK members) eligible parents (or primary caregivers) of children who fulfilled the criteria received an information letter from the BOSK inviting them to participate in the study. A stamped return-envelope, an informed consent form, information about the study, and a diary covering a 7-day period, were included. This study was conducted jointly with a quantitative study aimed to give a wide-ranging portrayal of the number, domains, and priority of needs expressed by parents using the Family Needs Inventory — Pediatric Rehabilitation [24, 25]. Parents (or primary caregivers) had the choice to refuse cooperation, to fill in the diary or the questionnaire, or both.

Parents (or primary caregivers) who did not respond to the first invitation received a reminder from the BOSK after three weeks.

### Data gathering

Participating parents (or primary caregivers) returned the completed diary and/or questionnaire as well as the informed consent form in the return envelope directly to the Centre for Data and Information Management (MEMIC) in Maastricht.

Solicited diaries, with optimum length between 1 and 2 weeks, provide a rich source of data and are often utilised to elicit specific information [18, 26, 27]. Participants were asked to complete the diary each day over a 7-day period, reflecting on issues that are of interest to the study — actions, challenges, and needs while supporting their child with a physical disability at home, at school, or in the community. A pilot study including fifteen parents (February 2012) revealed that the 7-day period solicited diary was feasible and took about 15 min per day to complete.

Participants used a printed diary (A4 booklet template) with worksheets for each day of the week, and an example of the worksheet with participant instructions. On each sheet columns were created for *Time of start of the activity*, *Activity the child was engaged in, Actions parents performed, Challenges parents came across, Needs parents experienced,* and *the possibility for additional remarks* (for an example of a spreadsheet see Table 1). Participants were asked to write down all activities their child did over 24 h, name the actions they did to facilitate those activities, and mention challenges and unmet needs they experienced. Next, an option was given for possible additional information.

**Table 1:** An example of the worksheet for one day of the week. Monday

	A	B	C	D	E
Time	Activity the child engages in	Do you support your child (sometimes) during the activity? If yes, how?	Do you face challenges/problems with this? If yes, which one?	What kind of support do you need?	Additional remarks
6.00-9.00					
9.00-12.00					
12.00-15.00					
15.00-18.00					
18.00-21.00					
21.00-24.00					
24.00-6.00					

### Data analysis

For the data-analysis of the diaries both deductive and inductive reasoning was used [28, 29]. Directed content analysis [23, 27], using deductive reasoning, was conducted to validate or conceptually extend the existing preliminary thematic framework as described above [17]. In this deductive analysis, the existing framework guided development of an initial coding and categorising scheme, and operational definitions for the codes were used [30]. Inductive reasoning was also used. In this way, potentially new major themes, categories and/or sub-categories could be identified from the data through the researcher’s careful examination and constant comparison [31].

After transcription of all diaries into a word-processing package, a coding scheme was developed and subsequently applied by means of manual coding. The first author (BP) prepared the coding scheme and the second author (AJHMB) inspected it to ensure congruence with the elements of the preliminary thematic framework. Then, the first author (BP) applied the identical coding and categorising scheme to all data using techniques of memoing, constant comparison, and questions. NVivo software (v.9, QSR International, Cambridge, MA) was used to organise the data during analysis. Two debriefing sessions took place between the user as co-researcher (BC) and the first author (BP) to discuss the findings leading to small changes in the wording of the codes of actions, challenges, and needs.

In this study Lincoln and Guba’s [32–34] four criteria (credibility, transferability, dependability, and confirmability) for evaluating interpretive research work were applied in accordance with Elo’s et al. [35] aspects of trustworthiness in relation to qualitative content analysis. Credibility was ensured with organising debriefing sessions between the first researcher (BP) and a co-researcher (BC) and by using random sampling. Transferability was guaranteed by passing information to the reader about the boundaries of the study and providing characteristics of the study population. Dependability is assured by reporting in detail the processes within this study, thereby enabling a future researcher to repeat the work, as is confirmability by reporting findings that are solely the result of the experiences and ideas of the participants.

## Results

In total, 51 diaries were returned. Three diaries were largely unfilled and one completely empty, and so these were excluded, leaving 47 eligible for the analysis. Participants were all biological parents — mainly mothers (92%) — who have a child with a disability who is between 4 and 12 years of age (for more characteristics see Table 2).

**Table 2:** Demographic characteristics of study participants

Family characteristics	
Mean/SD age participant (*n* = 47):	43 (5.4)
Relationship with the child	Number (%)
Mother	43 (92%)
Father	4 (8%)
Other	0 (0%)
Nationality of the respondent	Number (%)
Dutch	47 (100%)
Other	0 (0%)
Highest level of education of participant	Number (%)
Less than high school	1 (2%)
High school	4 (8%)
More than high school *(total Dutch female population =36%)*	42 (90%)
Family Type - child lives:	Number (%)
with 2 biol. parents	43 (92%)
with 1 biol. parent	4 (8%)
Child characteristics	
Mean/SD age child (*n* = 47):	7.9 (2.6)
Child nationality	Number (%)
Dutch	45 (96%)
Unknown	2 (4%)
Child gender	Number (%)
Female	22 (47%)
Male	25 (53%)
Child education	Number (%)
Regular	23 (50%)
Special	14 (30%)
Unknown	10 (20%)

**Fig. 2:**
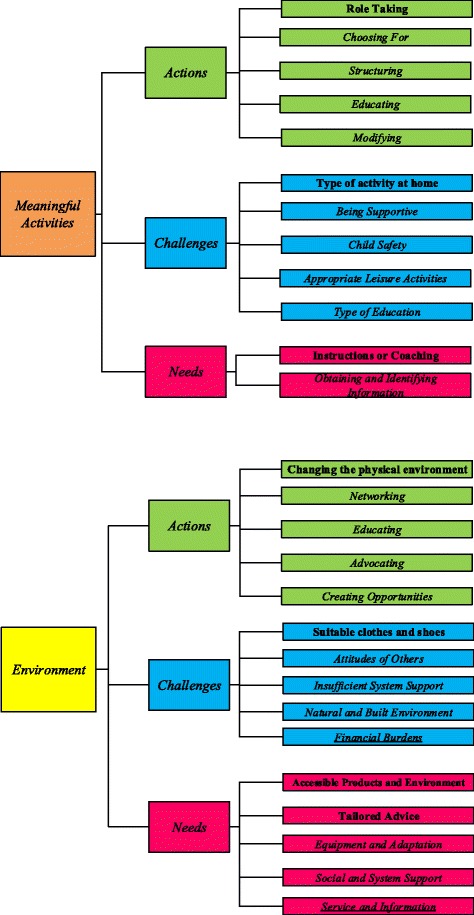
Preliminary framework of parent’s actions, challenges and needs based on the results of the diary study

In comparison with the original framework, two previously identified major themes, “parents enable and support performance of meaningful activities at home, at school and in the community” and “parents enable, change and use the environment”, remained the same. Additionally, the categories “actions”, “challenges”, and “needs” did not change. However, in all three categories new sub-categories were identified: two in the category actions, two in the category challenges, and three in the category needs. On the contrary, the previously named challenge regarding *financial burden*, and the need for *service and information* were not identified in the current analysis. Figure 2 portrays the presentation of all the major themes, categories, and sub-categories that were identified in both studies. Previously identified major themes with categories and sub-categories of the preliminary framework [17] that remained the same are marked “*italic”*; new sub-categories that emerged from the inductive analyses are marked **“bold”**. Two sub-categories that were not identified in this study are “underlined”.

### Major theme 1: Parents enable and support performance of meaningful activities at home, at school, and in the community

This theme concerns actions, challenges, and needs of parents while supporting their child with a physical disability to engage in meaningful activities at home, at school, or in the community.

#### Category: Actions to support meaningful activities

One new action was identified (**role taking**) and four previously identified actions (*choosing for, structuring, educating, and modifying*) emerged in this study.

The new action **“role taking”** implies how parents are taking up a new role that allows supporting meaningful activities of their child. Parents in this study act as a volunteer in scouting to help their child to complete tasks like cooking or assisting a swimming teacher. Parents also gave assistance to teachers at school during physical education.

The action *“choosing for”* refers to parents making choices regarding the kind of activities in which their child will be socially engaged. A number of examples were described in the diaries: deciding on joining a birthday party with peers without a disability, playing at a friend’s home, or playing outside the house with neighbours. Parents mentioned that they chose a team sport, such as judo or grass-hockey, as a leisure activity for their child to enable contact with peers without a disability. Parents also described their choice for doing “normal” family activities outside their house, such as visiting an open-air museum by bus or going to a playground.

*“Structuring”* describes the parents’ action to organise daily routines at home in such a way that their child can engage in meaningful activities. One parent explained the change of a daily routine of eating a warm evening meal at 6 p.m. to be in time for horse riding.

The action *“educating”* applies to teaching the child a new strategy to be able to participate in activities. Parents described this action with different examples, such as showing their child an easier way to use the computer; showing their child how to perform a domestic task such as preparing cutlery for seven people using a strategy to count aloud one, two, three…; and by using modelling while practicing with a flute. Examples related to school were also described, such as teaching their child how to reach his classroom independently and showing an alternative way of doing mathematics.

The action *“modifying”* stands for adaptations of activities to support the child’s independence and social interaction. One example in this study was about a family walk in a forest. A mother described that she walks next to her bicycle while her child with a physical disability sits on it. Another example is buying clothes with Velcro tape that enables a child to dress himself at school.

#### Category: Challenges while supporting meaningful activities

Parents reported several challenges that emerged while supporting their child’s meaningful activities. One new challenge (**choosing the right type of activity at home**) and four formerly identified challenges (*being supportive in a correct manner*, *coping with child safety*, *choosing the most appropriate leisure activities*, and *selecting the best type of education*) have been identified.

The challenge “**choosing the right type of activity at home”** refers to the struggle over how to decide what kind of activities their child can engage in with other children at home. An example is that parents go through great effort to find an appropriate activity suitable for their child to play together with a brother or sister without a disability.

The challenge “*being supportive in a correct manner”* is about deliberating whether the child is capable of doing an activity (at home or outside) alone and estimating what would be appropriate support. Several examples were given in this study. Parents explained that it is a dilemma during breakfast whether or not to cut bread; the child had the ability to do it alone but it might slow down the morning routine, resulting in being too late for school or the school-taxi. Other examples were parents struggling with whether they should tie their child’s shoes or to explain how to do it, and how much support should be given during bathing, showering, dressing, or cycling.

“*Coping with child safety”* is about parents having difficulties coping with situations with no parental control. Activities provoking these challenges were: learning how to make a fire at scouting, playing at a friend’s home, and going with another family to a playground.

The challenge *“choosing the most appropriate leisure activities”* indicates problems finding a leisure activity that fits the child’s abilities and brings a sense of accomplishment. Parents reported challenges finding suitable team sports and appropriate swimming lessons for their child.

*“Selecting the best type of education”* is a challenge parents also faced in this study. For example, one parent explained that it was difficult to make the choice for the type of education for their child, as they got dissimilar advice from different professionals.

#### Category: Needs while supporting meaningful activities

Parents provided information about unmet needs while supporting meaningful activities of their child. In this study one new need **(instructions or coaching**) emerged and one existing need (*identifying and obtaining information)* was confirmed.

The need **“instructions or coaching”** is about receiving written or verbal support. One parent wrote in the diary that having instructions or coaching in how to put their child into the car and how to put her in the seat would be helpful. Other parents expressed a need for instructions to support independence during meals. One parent showed a need for coaching on how they can support their child while playing in a play garden. In addition, parents expressed a need for coaching in understanding laws and regulations (e.g. the ‘Social Support Act’ — WMO) or in choosing a new type of educational program.

*“Identifying and obtaining information”* is a need for more information about meaningful activities. For example, one parent expressed the need for an overview of suitable leisure activities for children with a physical disability.

### Major theme 2: Parents enable, change, and use the environment

This theme relates to actions, challenges, and needs of parents while using, enabling, and changing the social and physical environment at home, at school, and in the community to make participation of their child with a physical disability possible.

#### Category: Actions to enable, change, and use the environment

One new action (**changing the physical environment**) was identified and four previously discovered actions (*networking, educating, advocating, and creating opportunities*) were confirmed.

The new action **“changing the physical environment”** demonstrates how parents take the initiative to change the physical environment into a more suitable one to support their child’s participation. One example of such action is an adaptation of a bathroom at home to aid the child in becoming independent. Another example of this kind of action is that some parents made a school entrance accessible to their child.

“*Networking*” refers to the establishing of connections with people with similar experiences. Parents in this study explained their attempts to find people through their social network to connect and share experiences, and their attempts to connect with people through social media, like forums.

The action “*educating*” is defined as the giving of instructions to others on how to support the participation of their child. Examples in this study are parents educating employees of the day care centre or educating a judo trainer to optimise assistance of their children.

“*Advocating*” refers to the competing of resources, supports, and services within the system. One parent reported in the diary about her way of going up against a taxi service to make sure their child would get extra support and be taken on board as a passenger.

“*Creating opportunities*” means the creation of events by parents in order to shape opportunities for their child to get acquainted with other children. One example in this study concerns parents organising a “get together” meeting where their child could connect to other children. Additionally, this action is also about making and keeping connections at school. One parent described in the diary that she organised extra after-school lessons to make sure her child will be able to stay in the same group of children next year.

#### Category: Challenges to enable, change, and use the environment

One new challenge (**customised products**) and three previously recognised challenges (*attitudes of others, insufficient system support,* and *barriers in the natural and physical environment*) emerged during the analysis.

“**Customised products”** pertains to a new challenge faced by many parents in this study. Several examples were described about difficulties in trying to find appropriate clothes that support independence and the challenge of finding fitting shoes, mainly for children that need to wear a splint. Parents also noted that it is difficult to find shoes with Velcro tape, particularly in bigger sizes.

The “*attitudes of others*” refers to the experience of parents facing negative attitudes from other children or adults towards their child with a physical disability. Examples in this study are negative attitudes of friends during play activities and negative remarks about disability from friends’ parents. Similar attitudes were experienced with professional services, like taxi service employees (drivers) reacting negatively towards a child with a disability and not being willing to help a child to get in and out of the taxi.

The challenge “*insufficient system support*” concerns unsupportive social structures. Parents in this study described that teachers and sports instructors lack knowledge of how to support children with a physical disability at school and during swimming lessons. Parents mentioned that support assistants (paid from personal budget funded by the Dutch Exceptional Medical Expenses Act — AWBZ) are not always capable of supporting their child, such as while eating a meal or engaging in play activities with their child at home. Others wrote that it is difficult to get a support assistant between 6 a.m. and 8 a.m. One parent described the complication of getting assistance for an empty wheelchair tire when visiting a museum.

*A “barrier in the natural and built environment”* refers to the physical accessibility of buildings and public places. Parents’ examples of these challenges concern the built environment: non-user-friendly public toilets, as at the Zoo; inaccessible walking paths in an open-air museum; and family cars that are unable to fit in a special swing-auto chair or a wheelchair. Similar challenges were described for the natural environment, like the impossibility to use walking paths in the forest with a wheelchair.

#### Category: Needs to enable, change, and use the environment

Two new needs (**accessible products and environments** and **tailored advice about equipment, devices, and adaptations**) and two previously identified needs (*equipment and adaptations, social and system support*) emerged.

“**Accessible products and environments**” shows a new need for products and environments designed to be usable by all people. Parents gave examples of this need in relation to the built environment (e.g. shops and other public places, houses, playgrounds) but also to shoes and clothes.

**“Tailored advice about equipment, devices, and adaptations”** illustrates a need for personalised advice about equipment, devices, and adaptations. Examples from this study describe a need for advice about adaptations in the house or in the day care centre. Furthermore, parents expressed a need for advice about the availability and appropriateness of devices for their child and about suitable clothes, shoes, and adaptive cutlery.

“*Equipment and adaptations*” refers to the need for equipment that is designed to support independence and participation in activities. Examples from this study demonstrate a need for equipment to support and facilitate independence while eating. Furthermore, parents expressed a need for equipment that can be used in a normal car, a need for easy-to-modify adaptations for a toilet or a shower, and a safe bicycle seat for bigger children.

“*Social and system support*” refers to the needs of parents for more expansive social networks to enable their child’s participation. In this study parents’ examples referred to extra support from grandparents and support assistants to bring their child to leisure activities, assistance during physical education at school or during church services, engagement in play activities at home or in the play garden, and to do educational activities on a computer.

## Discussion

The purpose of this study is to gain insight into parents’ own daily actions, challenges, and needs while supporting their child with a physical disability at home, at school, and in the community. In one week, on every single day, all participating parents described several efforts to enhance the participation of their children with a physical disability by using, enabling, or changing the social and physical environment, or by supporting their child to perform or engage in meaningful activities. Fascinatingly, those actions were primarily a result of challenges caused by restrictions in social and physical environments. Needs described by parents notably spotlight environmental aspects, like a need for environments designed for all people.

Additionally, this study intended to refine the existing preliminary thematic framework arising from a scoping review [17]. Two major themes, all categories and sub-categories, except two, of the preliminary thematic framework [17] were consistent with the actions, challenges, and needs mentioned by the parents in this study. One challenge regarding *financial burden*, and one need for *service and information* were not identified in the analysis.

However, the analysis reveals two new actions (*role taking*, *changing the physical environment*), two new challenges (*choosing the right type of activity at home*, *customised products*), and three new needs (*instructions and coaching; accessible products and environments; tailored advice about equipment, devices, and adaptations*).

The previously named challenge regarding *financial burden*, and the need for *service and information* were not identified in the current study; this might be related with socio-economic status and level of education of our sample. However, at this point this is a speculation.

The previous scoping review [17] showed that parallels exist between the parents actions, challenges, and needs described in the preliminary framework for children with a physical disability and studies done with parents of children with Down syndrome, young people with epilepsy, and young adults with a physical disability [36–38]. Studies and policy reports from the Netherlands support the newly found actions, challenges, and needs related to environment. For example, free access to public buildings and places for all citizens in the Netherlands was not taken into the Equal Opportunities Act; there is no obligation to guarantee access for persons with a disability [39]. Consequently, only 29% of people with a severe physical disability in the Netherlands can enter shops [40]. Parents in this study expressed a new need for *“accessible products and environments”*, like playgrounds, and showed their own initiatives to change the physical environment into a more suitable one to support their child’s participation (action: *“changing the physical environment”)*. In the Netherlands there are no specific regulations for leisure and sports clubs concerning children with a disability [41]. The inclusion of children with disabilities in mainstream primary education has been arranged by law in August 2014 [42]. Therefore, it might be valuable to all educators to construct learning experiences that are meaningful for all young children, including those with diverse abilities, by applying universal design principles for learning [43].

The 47 included diaries indicate that parents of children with a physical disability carry out many actions, face numerous challenges, and have several unmet needs while supporting their child’s participation at home, at school, and in the community. It is interesting that parents’ actions, challenges, and needs are mainly directed towards the social or/and physical environment. Therefore, paediatric rehabilitation using FCS may need to address the importance and the impact of the environment on the participation of a child with a physical disability, rather than only focus on the child’s abilities. Care professionals might need to ask themselves whether the real world of children with a physical disability and their parents is central in their approach and whether they involve the knowledge of parents in shared-decision making.

In order to effectively support parents while enabling the participation of their children in daily life, tailored approaches are compulsory. These approaches may also contribute to stress reduction and better health and well-being of parents. Raina et al. [44] found that health and well-being of parents of children with cerebral palsy seem strongly influenced by child disability; Parkes et al. [45] showed that a quarter of parents of children with cerebral palsy experience very high stress. However, any approach needs to be based on parents’ wishes for support. The goal of most qualitative studies is not to generalize but rather to provide a rich, contextualized understanding of some aspect of human experience [46]; however, the analysis showed that several actions, challenges and needs of the Dutch parents resemble with the actions, challenges and needs from other studies in Europe or outside [17]. Future research into the meaning parents ascribe to their experiences regarding actions, challenges, and needs while supporting participation of their child in different cultural contexts is warranted.

### Study limitations

Some possible limitations in this study are noteworthy to discuss. Selection bias may have occurred as we used the database of the Dutch Association of People with Disabilities and their Parents (BOSK) to draw our participants from, and only 9% of eligible parents decided to participate. The participating parents, 92% mothers, had a higher educational level than average in the Netherlands, and, considering their membership in BOSK, may have been parents who are very involved and motivated in enhancing the participation of their child with a physical disability. However, the data set is rich in nature and there is a large variety among data; some parents reported more actions, challenges, and needs at home, and others at school or in the community. Further, mothers are usually the respondents in other similar studies, like Almasri et al. [47]. They are known as caretakers of children with a disability, and are therefore most involved in enhancing the participation of their child. In a study about parents of children with intellectual disabilities, Rowbotham et al. [48] found that mothers undertake more daily care-giving tasks than fathers, but the range of tasks is similar. However, it is unclear how such differences influence actions, challenges, and needs of fathers and mothers. Additionally, it can be argued whether data collection of just one week is sufficient. As the optimal length for this type of study is one to two weeks [27] and the data received were rich in nature, the period of time probably has not been a limitation.

## Conclusions

Actions, challenges, and needs of Dutch parents’ of children with a physical disability are mainly directed towards the social or/and physical environment. The presented thematic framework can offer practitioners knowledge of how to support parents and promotes relevance for future research investigation. With the intention of supporting parents, further work is necessary to supply tailored approaches.

## Abbreviations

BOSK: the Dutch Association of People with Disabilities and their Parents
FCS: Family-centered services
MEMIC: Centre for Data and Information Management in Maastricht
